# Fuelling Recovery: Is There a Role for Radiation Therapists in Optimising Nutrition for Women With Breast Cancer?

**DOI:** 10.1002/jmrs.874

**Published:** 2025-03-27

**Authors:** Laura Feighan, Lesley MacDonald‐Wicks, Robin Callister, Yolanda Surjan

**Affiliations:** ^1^ Global Centre for Research and Training in Radiation Oncology, School of Health Sciences, College of Health, Medicine, and Wellbeing The University of Newcastle Callaghan New South Wales Australia; ^2^ School of Health Sciences, College of Health, Medicine, and Wellbeing The University of Newcastle Callaghan New South Wales Australia; ^3^ School of Biomedical Sciences and Pharmacy, College of Health, Medicine, and Wellbeing The University of Newcastle Callaghan New South Wales Australia

**Keywords:** breast cancer, nutrition, radiation therapists, radiation therapy, unmet needs

## Abstract

**Introduction:**

Women with breast cancer receiving radiation therapy (RT) can experience treatment side effects and compromised quality of life. The quality of a person's diet can play a vital role in preventing cancer and other chronic diseases. Despite this, studies show many Australians do not meet the recommended guidelines for consuming a quality diet. Currently, women with breast cancer receiving RT are not routinely provided nutritional support, highlighting a possible gap in the comprehensive care of this population. This study aims to explore the dietary practices of women with breast cancer receiving RT and their perceptions of nutritional support during treatment. Furthermore, an investigation of the radiation therapists' role in providing nutritional support is considered.

**Methods:**

A cross‐sectional study design was implemented via an online survey. The survey was offered to women with breast cancer receiving RT in Australia's Capital Territory, New South Wales and Queensland regions. The survey comprised 70 questions focused on exercise, nutritional habits and overall health. The Short Dietary Questions were used specifically for the nutrition‐based questions.

**Results:**

Ninety women completed the survey; none met all recommended dietary guidelines, with only 33% consuming recommended fruit servings and 12% meeting vegetable requirements daily. While half the women reported receiving dietary guidance during RT, the content and quality of advice are unclear.

**Conclusion:**

This study highlights both the dietary patterns of women with breast cancer receiving RT and their unmet needs for nutritional guidance. While nutritional support is crucial for patient wellbeing during cancer treatment, further research is needed to determine optimal approaches for diet education delivery. Specifically, investigation into how radiation therapists can effectively integrate evidence‐based nutritional support into their practice to improve patient care.

## Graphical abstract

A study of 90 Australian women receiving breast cancer radiation therapy found none met dietary guidelines, with only 33% consuming enough fruit and 12% enough vegetables daily, indicating a crucial need for improvement in nutritional support during treatment.

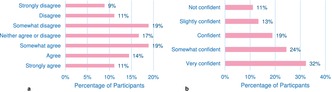

## 1 Introduction

A person's diet, which refers to the food and beverages they consume, is an important aspect of maintaining good health [1]. To ensure optimal health, it is necessary to have a well‐balanced diet that includes a variety of foods, providing essential nutrients in appropriate proportions [2]. The nutritional value and composition of an individual's diet, which is the ‘diet quality’, play an important role in preventing chronic illnesses such as cancer [3]. It is recommended to consume nutrient‐rich foods such as fruits, vegetables, whole grains, lean protein and healthy fats while limiting the intake of processed foods, added sugars and unhealthy fats (known as discretionary foods). By adopting such a high‐quality diet, individuals can ensure adequate nutrient intake while reducing the risk of nutrient deficiencies [4].

Studies show that many Australian women between the ages of 18 and 70 years do not meet the recommended daily servings of the five main food groups [1]. Additionally, a significant portion of their energy (kilojoule) intake comes from discretionary foods, specifically, soft drinks, fruit juices and cakes, consumed in more than recommended amounts [1]. This low‐quality diet (alongside a lack of physical activity, heightened stress and smoking) is associated with breast cancer being ranked the most commonly diagnosed cancer in women worldwide [5, 6]. Furthermore, Ng et al. report that nearly 90% of Australians aged 45 years and over who are diagnosed with cancer have at least one other chronic illness, such as cardiovascular disease and/or hypertension, both of which may also be due to a poor diet [7, 8].

Treatment options for breast cancer, such as surgery (lumpectomy or mastectomy), chemotherapy, radiation therapy (RT) and targeted therapy, can cause side effects [9]. RT specifically may cause skin reactions, radiation‐induced fatigue and lymphoedema [10]. These challenges can also lead to added nutritional depletion. The relationship between diet quality and mental health has been investigated, particularly anxiety, depression and stress, which all correlate with an increased tendency to eat discretionary foods and eat impulsively [11, 12].

Patients with cancer diagnoses of the oesophagus, gastrointestinal tract (GIT) and head and neck (H&N) typically require diet management during RT [13]. This is due to the toxicities these anatomical sites experience as a result of RT treatment (nausea, vomiting and dysphagia) [14]. These side effects can cause severe nutritional deficiency and weight loss that can instigate hospitalisation and disruption to the treatment regimen. To ensure prompt nutritional screening/assessment and management, the contribution of a multidisciplinary team (such as dietitians, speech pathologists and radiation oncology nurses) is integrated [15].

Evidence‐Based Practice Guidelines for the Nutritional Management of Patients Receiving Radiation Therapy by Isenring et al. state that appropriate access to care for all patients receiving RT to the oesophagus, GIT and H&N should include referral to a dietitian and/or nutrition support. This includes an assessment with an evidence‐based nutrition assessment tool such as the Subjective Global Assessment (SGA) [16]. The Guidelines aim to ensure that quality of life is maintained, symptoms are managed and weight loss is reduced [17].

Currently, dietary guidance for women undergoing RT for breast cancer is limited, despite evidence reporting nutritional support benefits for patients with various cancer diagnoses. There is a lack of information on how dietary recommendations might enhance the RT experience and recovery process for women with breast cancer specifically. Furthermore, as radiation therapists see patients for daily appointments throughout their treatment regimen, they are well placed to provide consistent supportive care (including dietary guidance). Despite this, there is currently a gap in educational preparation and professional development opportunities to support the delivery of this care. Notably, a qualitative study by Halkett et al. conducted semi‐structured interviews with 34 patients receiving RT for breast cancer to evaluate patients' perspectives on the role of radiation therapists. It was found that patients felt supported during treatment when radiation therapists provided information related to their overall care [18, 19, 20]. Addressing educational paucity via the integration of nutritional training could improve radiation therapists' capability and confidence in providing nutritional support and ultimately strengthen patients' treatment experience and recovery.

This study seeks to examine the eating habits of women receiving breast cancer treatment and assess their perceived need for additional nutritional guidance during this period. Furthermore, it aims to investigate how radiation therapists can effectively integrate dietary support into their broader role of addressing unmet supportive care needs for women with breast cancer.

## 2 Methods

### 2.1 Settings and Participants

A cross‐sectional study design was utilised via an online survey. The survey, with a focus on nutrition and exercise, was designed to gather information on the experiences of women undergoing treatment for breast cancer (see Supporting Information). To be eligible, participants had to be female, over 18 years old and currently undergoing RT treatment, having received at least five fractions but not yet completed treatment. Participation in the survey was voluntary and anonymous.

Fifteen Australian radiation oncology (RO) departments were invited to participate in the study and assist in recruiting participants. The RO staff, including nurses, radiation therapists and radiation oncologists, notified patients about the survey and provided designated tablets/computers to complete the survey on‐site, either before or after a fraction of treatment. The survey was conducted between September 2023 and March 2024 in three Australian regions (Australian Capital Territory, New South Wales and Queensland). The study received ethical approval from the Hunter New England Human Research Ethics Committee (2019/ETH00345).

### 2.2 Survey

The survey was conducted online via QuestionPro (www.questionpro.com), with the guidance of RO staff. It comprised 70 questions, both multiple‐choice and open‐ended responses. The survey was divided into six sections: demographics, general health, smoking and alcohol, physical activity/exercise habits, diet habits and exercise and nutrition as interventions.

This manuscript explores responses from participants related to diet habits and knowledge (it is intended the physical activity/exercise responses will be reported in a future manuscript). In particular, the section investigating diet habits contained the Short Dietary Questions (SDQs) from the 1995 National Nutrition Survey [21]. The SDQs are a brief tool used to gather data on core food group frequency of intake and eating frequency. One of the key advantages of using SDQs is that they provide specific and potentially valuable insights into the food and nutrition trends of a population [22]. The use of SDQs facilitated the collection of significant nutritional data without imposing a burdensome survey on the participants. Several questions in the general health section were derived from the Exercise and Nutrition Routine Improving Cancer Health (ENRICH) Program study, which investigated a novel lifestyle intervention to improve adult cancer survivors' health behaviours [23]. The smoking and alcohol section used questions from a study by Bryant et al. [24] that examined the prevalence and clustering of six health risk behaviours (smoking, alcohol, inadequate sun protection, physical inactivity and inadequate fruit and vegetable consumption) among severely disadvantaged individuals. The study asked questions regarding alcohol from the AUDIT Alcohol Consumption Questions (AUDIT‐C) [25]. These validated surveys were used to enable comparisons with existing literature and to establish baseline data for future research.

### 2.3 Data Analysis

Data from the responses were exported from QuestionPro to Microsoft Excel for analysis. While the SDQs effectively captured food trends within our cohort, the tool's design does not support a robust statistical covariate analysis. Therefore, responses are reported simply as counts and percentages.

## 3 Results

### 3.1 Participants

The survey was completed by 90 women in Australia. Most women (57%) were between 45 and 64 years old. Cancer treatment included the majority (80%) of women undergoing a lumpectomy, and 39% undergoing chemotherapy before RT. Furthermore, 13% of women received targeted (molecular) therapy in addition to RT when completing the survey (Table 1). Most women (58%) were employed (part‐time, full‐time or casual); however, 41% either decreased their hours or stopped working entirely during treatment. When asked about caring responsibilities separately from employment status, 19% reported caring for children, parents or spouses. Radiation therapy prescriptions ranged, but most (59%) were prescribed 40 Gray in 15 fractions, 16% were prescribed 50 Gray in 25 fractions and 13% were prescribed 42.5 Gray in 16 fractions. On average, participants had completed (mean + SD = 11 + 4) fractions when they completed the survey.

**TABLE 1:** Demographic characteristics of respondents. Data are presented as counts and percentages for the 90 respondents.

Characteristics	Responses *n* (%)
Age (years)	
< 45	10 (11%)
45–74	71 (79%)
> 75	9 (10%)
Marital status	
Married—put de facto underneath	51 (57%)
Widowed	6 (7%)
Divorced/Separated	14 (16%)
De Facto	9 (10%)
Never married	10 (11%)
Employment	
Full‐time	24 (27%)
Part‐time	14 (16%)
Casual	5 (6%)
Self‐employed	9 (10%)
Maternity leave	1 (1%)
Parent/Carer	3 (3%)
Unemployed/Looking for work	5 (6%)
Retired	29 (32%)
Cancer treatment (in addition to RT)	
Lumpectomy	74 (80%)
Mastectomy	18 (20%)
Axillary node dissection	21 (23%)
Breast reconstruction after mastectomy	3 (2%)
Breast reconstruction planned for a later date	9 (10%)
Chemotherapy	35 (39%)
Targeted therapy	12 (13%)

Comorbidities were experienced by most women, with hypertension (32%), high cholesterol (29%) and mental health conditions (anxiety, depression or posttraumatic stress disorder (PTSD)) (29%) being the most widely reported. Notably, 50% of women had two or more comorbidities, with 22%, 12%, 8% and 7% experiencing two, three, four and five or more, respectively (Table 2). Just 3% experienced lymphoedema, all reported as ‘mild’ and not requiring a compression garment. Participants' average body mass index (BMI) was 27.8 kg.m^−2^ with 2% in the underweight range, 28% in the healthy range, 44% in the overweight and 26% in the obese category.

**TABLE 2:** Comorbidities of the respondents. Data are presented as counts and percentages of the 90 respondents.

Comorbidities for each participant	Responses *n* (%)
Hypertension	29 (32%)
High cholesterol	26 (29%)
Liver/kidney condition	2 (2%)
Diabetes; Type 2	8 (9%)
Heart condition	3 (3%)
Stroke	2 (2%)
Lung condition	15 (17%)
Musculoskeletal disorder	11 (12%)
Arthritis	17 (19%)
Stomach or duodenal ulcer	1 (1%)
Chronic headaches/migraine	13 (14%)
Anxiety, depression and PTSD	26 (29%)
Other (ellipsis, osteoporosis, hiatus hernia, hypothyroidism, obsessive‐compulsive disorder, endometriosis, polycystic ovaries and allergic rhinitis)	8 (9%)

Most women (89%) did not smoke tobacco products, although 44% had smoked at least 100 cigarettes throughout their lives. The consumption of alcohol varied, with the majority (53%) reporting they never consumed alcohol or only had a drink containing alcohol monthly or less.

### 3.2 Diet

The Australian Guide to Healthy Eating (AGTHE) Guideline #2 set by the National Health and Medical Research Council (NHMRC) (see Table 3) recommends consuming a variety of foods from the core food groups every day (vegetables, fruit, grain, lean meats and dairy) [26].

**TABLE 3:** National Health and Medical Research Council, Australian Guide to Healthy Eating for women between 18 and 75+ years [26].

Fruit (150 g per serving): 2 serves per day
Vegetables (75 g per serving): 5 serves per day
Grain: 3 serves per day (70+ years of age), 4 serves per day (51–70 years of age) and 6 serves per day (19–50 years of age)
Lean meats (cooked beef and lamb), poultry, fish, eggs, tofu, nuts/seeds and beans: 2 serves per day (70+ years of age), 2 serves per day (51–70 years of age), 2.5 serves per day (19–50 years of age)
Recommend reduced‐fat milk

For meal frequency, most women (63%) reported having something to eat two to four times per day. Additionally, a large percentage of women (73%) consumed breakfast five or more days per week (Table 4). Only 12% reported eating the recommended five servings of vegetables daily, while 26% consumed four. Regarding fruit consumption, 33% reported consuming the recommended two servings daily.

**TABLE 4:** Respondents' diet characteristics.

Diet characteristics	Responses *n* (%)	Met AGTHE guidelines
Frequency of meals per day (including snacks)		
Once	2 (2%)	
2–4 times	57 (63%)	
5–6 times	22 (24%)	
7 or more times	4 (4%)	
Unsure/varied	5 (6%)	
Breakfast consumption (days per week)		
Rarely or never	8 (9%)	
1–2 days	6 (7%)	
3–4 days	10 (11%)	
5 or more days	66 (73%)	
Vegetable consumption (75 g serves per day)		
1 serve	14 (16%)	
2 serves	21 (23%)	
3 serves	15 (17%)	
4 serves	23 (26%)	
5 serves	11 (12%)	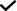
6 serves or more	6 (7%)	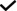
Salad consumption (times per week)		
Less than once per week	10 (11%)	
1–2 times	33 (37%)	
3–7 times	41 (46%)	
7 or more times	6 (7%)	
Cooked vegetable consumption (times per week)		
1–2 times	25 (28%)	
3–7 times	54 (60%)	
7 or more times	11 (12%)	
Potato consumption (times per week)		
Less than once per week	28 (31%)	
1–2 times	36 (40%)	
3–7 times	25 (28%)	
7 or more times	1 (1%)	
Chips (French fries, wedges, fried potato or crips) consumption (times per week)		
Less than once per week	42 (47%)	
1–2 times	41 (46%)	
3–7 times	7 (8%)	
Fruit consumption (125 g serves per day)		
1 serve	18 (20%)	
2 serves	30 (33%)	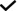
3 serves	11 (12%)	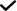
4 serves	9 (10%)	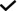
5 serves	8 (9%)	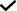
6 serves or more	10 (11%)	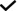
I don't eat fruit	4 (4%)	
Fruit juice consumption (times per week)		
Less than once per week	60 (67%)	
1–2 times	13 (14%)	
3–7 times	12 (12%)	
7 or more times	5 (6%)	
Bread consumption (times per day)		
Less than once per day	41 (46%)	
1–2 times	39 (43%)	
2–4 times	7 (8%)	
4 or more times	3 (3%)	
Bread type preferences		
White	19 (21%)	
Rye	2 (2%)	
Whole wheat	3 (3%)	
Brown	1 (1%)	
Wholemeal	23 (26%)	
Wholegrain	32 (36%)	
Flatbread/wraps	3 (3%)	
High fibre white	7 (8%)	
Pasta, rice and noodles consumption (times per week)		
Rarely or never	17 (19%)	
1–2 times	55 (61%)	
3–7 times	17 (19%)	
7 or more times	1 (1%)	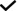
Meat products (sausages, frankfurters, devon, salami, meat pies bacon or ham) consumption (times per week)		
Rarely or never	40 (44%)	
1–2 times	43 (48%)	
3–7 times	7 (8%)	
Red meat (beef, lamb and pork) consumption (times per week)		
Rarely or never	16 (18%)	
1–2 times	46 (51%)	
3–7 times	27 (30%)	
7 or more times	1 (1%)	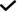
Milk type preferences		
Whole milk	36 (47%)	
Low/reduced fat	22 (29%)	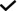
Skim	5 (7%)	
Nondairy	13 (17%)	
Sweets (biscuits, cakes, pastries, confectionary and sugar‐sweetened soft drinks/cordials) consumption (times per week)		
Rarely or never	20 (22%)	
1–2 times	39 (43%)	
3–7 times	26 (29%)	
7 or more times	5 (6%)	

*Note:* For evaluation purposes, an approximation was made due to slightly different serving sizes and frequencies in the AGTHE and SDQs.

Abbreviation: *Australian Guide to Healthy Eating* (AGTHE).

Wholegrain (36%) and wholemeal (26%) bread were the preferred choices. A substantial portion of women (46%) reported consuming bread less than once per day. More than half of the participants (61%) reported consuming pasta, rice, noodles and other cooked cereals once to twice weekly. Most women (47%) selected whole milk as their preferred choice, and only 29% reported reduced fat as their preferred option.

Approximately half of the participants (51%) reported consuming red meat (beef, lamb and pork) one to two times per week, and 48% reported consuming sausages, frankfurters, devon, salami, meat pies, bacon or ham one to two times per week.

The AGTHE Guideline #3 recommends limiting the intake of noncore foods containing saturated fat, added salt, added sugars and alcohol. The consumption of chips (French fries, wedges, fried potatoes or crisps) and sweets (biscuits, cakes, pastries, confectionery and sugar‐sweetened soft drinks or cordials) was also investigated. It was observed that chips were consumed less than once per week and sweets were consumed one to two times per week by most women (48% and 38%, respectively).

### 3.3 Participant Perceptions of Diet Habits During Radiation Therapy

**FIGURE 1:**
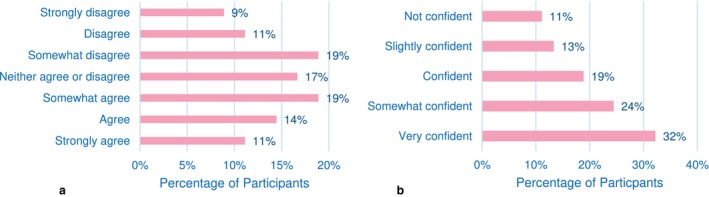
(a) Women's perceptions on receiving sufficient information regarding diet and nutrition during their RT treatment. (b) Women's perceptions of confidence in maintaining a healthy diet during RT treatment.

Participants had varying opinions regarding the adequacy of the nutrition and diet information they received during RT treatment (Figure 1a). A similar proportion of women agreed (44%) or disagreed (39%) to some extent that they were provided with sufficient information.

There is a wide range of confidence levels in maintaining a healthy diet (Figure 1b), while the majority (32%) reported high confidence, some were only ‘slightly confident’ (13%) or ‘not confident’ (11%).

Furthermore, a proportion of women (80%) responded ‘yes’ to participating in an exercise and nutrition intervention, if it were offered to them during their RT treatment. Statements such as: ‘So I know I'm doing the correct thing, as I've been told different [things] and it gets confusing*’* were expressed by women regarding why they would participate in an intervention.

### 3.4 General Health

**FIGURE 2:**
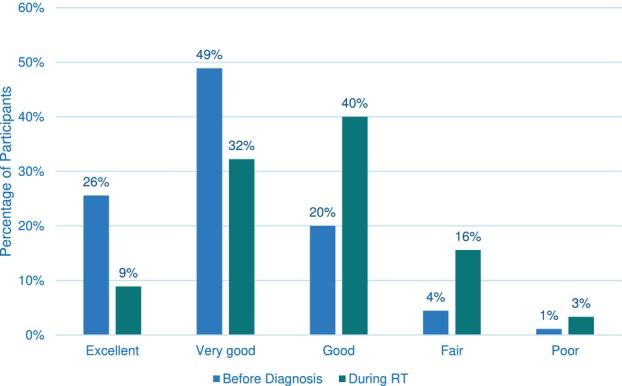
Women's perception of their general health before cancer diagnosis and during RT.

**FIGURE 3:**
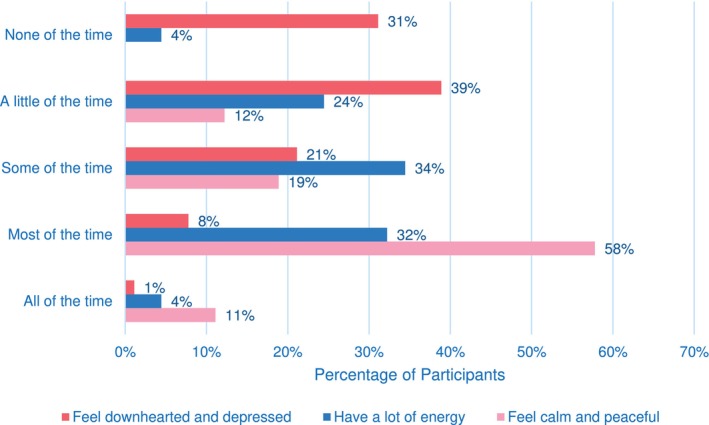
Women's responses to the amount of time they felt each of the statements regarding their physical and emotional health during RT.

Women were asked to rate their general health (from excellent to poor) at two different points—before their cancer diagnosis and during RT treatment (Figure 2). Almost half of the women (49%) perceived their health as ‘very good’ before diagnosis, whereas during RT, 40% perceived their health as ‘good’. Figure 3 provides insights into women's emotional and physical health perceptions. Women reported feeling calm ‘most of the time’ (58%), having a lot of energy ‘some of the time’ (34%) and experiencing a ‘little’ depression (39%). A proportion of women (56%) reported being limited by their physical health when engaging in moderate activities.

## 4 Discussion

This study explored the dietary habits of women diagnosed with breast cancer undergoing RT. The study found that none of the 90 participants met the recommended dietary guidelines, suggesting that these women were not consuming a quality diet. Additionally, some women reported a lack of confidence in maintaining a healthy diet during RT treatment. Despite it being plausible that nutritional assessment and guidance traditionally focused on the oesophagus, GIT and H&N, this study indicates that women with breast cancer receiving RT could benefit from similar considerations.

The Australian Bureau of Statistics reported on results from a National Health Survey (NHS) completed by 10,083 female participants (18 years and over) [27]. The daily consumption of fruit and vegetables was included, which is comparable to our study. The AGTHE guidelines suggest a daily fruit consumption of two servings, which only 33% of participants in both studies met. Furthermore, daily vegetable consumption of five servings was met by only a very small portion from each group (12%; our study, 7%; NHS) [26, 27]. These results suggest that the dietary habits of our sample of women are consistent with those of the wider female population.

### 4.1 Patient Perspectives

Our study's findings align with larger issues regarding nutritional support for individuals with cancer. The absence of dietary guidance for women with breast cancer reflects a broader problem in healthcare education and practice. The majority of women in our study (80%) reported an interest in participating in nutritional interventions. Despite women with breast cancer attending cancer care departments daily and interacting with health professionals such as radiation therapists regularly, it raises the question of why radiation therapists are not providing care and advice on nutrition. This gap in care is further highlighted by the current lack of education for radiation therapists in this area.

According to the interview results of 20 American patients with cancer, by Corr et al., most (*n* = 15–18) were rarely or never asked about their diet by their oncologist. Likewise, 15 participants reported that their oncologist seemed only somewhat prepared to address their diet and nutrition queries. This correlates with results from our study, where many participants (39%) disagree (to some extent) that they received adequate diet information during RT. It was also reported from a survey completed by 50 participants (as part of the Corr et al.'s study) that 28% and 46% of participants perceive nutrition to be an ‘extremely’ and ‘very’ important aspect of their cancer treatment, respectively [28]. Relatedly, Chou et al. identified unmet supportive care needs of women with breast cancer. Of 1129 participants, both psychosocial and nutritional domains (40.4% and 28.4%, respectively) were considered the most unmet supportive care needs of survivors [29]. Moreover, a comprehensive systematic review by Harrison et al. examining 94 studies on unmet supportive care needs in cancer patients revealed that the highest levels of unmet needs across most domains were consistently identified during the treatment phase [30]. This finding underscores the critical importance of addressing supportive care, including nutritional support, particularly while patients are undergoing active treatment.

### 4.2 Implications for Healthcare Practice

While patient perspectives are vital, it is equally important to consider healthcare provider experiences and perceptions. Nutrition and diet are not commonly studied subjects in undergraduate or postgraduate healthcare training (outside of degrees or courses specifically related to nutrition and dietetics) [31, 32]. Despite this, nutrition is considered an important element of supportive care in oncology [33]. Furthermore, it is a continually reported unmet need of people during active cancer treatment and survivorship; a scoping review by Johnston et al. discovered that patients often do not receive the necessary dietary support required to meet their needs and preferences [34]. This gap in nutritional support is further evidenced by prior investigations by the current authors. We surveyed 76 radiation oncology practitioners (radiation oncologists, radiation therapists and nurses) regarding their confidence in providing nutritional advice to their patients with breast cancer. It was found that only 13% always offered dietary guidance to their patients. Moreover, up to 59% and 38% agreed they would ‘definitely’ or ‘probably’ participate in training regarding providing nutritional support, respectively [35].

This lack of confidence in providing nutritional advice is not limited to one healthcare setting or country. In a Canadian study, Erlich et al. surveyed 137 radiation therapists to investigate their ability to recognise patients who require dietary support during RT and provide intervention. It was reported that 31% of radiation therapists routinely referred patients to dietitians; however, 15% reported not routinely referring (for sites such as GI and H&N). Although breast cancer was treated at the study's clinic, it was not reported if radiation therapists identified a need for dietary support among this cohort. Furthermore, 91% of radiation therapist respondents indicated an interest in receiving training in recognising dietary support needs in their patients [36]. Likewise, a qualitative study by Pallin et al. reports results of semi‐structured interviews with 15 radiation therapists in the United Kingdom regarding opinions on delivering health behaviour advice to patients living with and beyond cancer. It was identified that the delivery of advice was matched by knowledge. Specifically, for diet promotion, participants felt knowledge was a barrier [37].

### 4.3 Future Directions

These results collectively highlight a recurring theme of inadequate dietary support for women with breast cancer during RT, despite the recognised importance of nutrition in cancer care. The discrepancy between patient needs and current practices suggests an opportunity for improved nutritional guidance and support in breast cancer treatment. Given the regular connections between radiation therapists and patients, there may be potential for improving nutritional support within the existing treatment setting. Radiation therapists are well positioned to evaluate patients and offer prompt access to dietary information or make referrals to allied health professionals (such as dietitians) as needed [18]. However, as per the previous findings, radiation therapists and other radiation oncology practitioners often lack both consistent nutritional support practices and confidence in their nutrition knowledge.

To address the paucity of knowledge of nutritional best practices, radiation therapists have expressed interest in online postgraduate training for diet and lifestyle behaviour promotion, which could help overcome time constraints while ensuring continuous professional development [37, 38]. Furthermore, a narrative review by Konstantinidis et al. found that radiation therapists consider e‐learning an equally or more appealing option than traditional education methods for lifelong training [39]. Supporting the viability of this approach, a mixed methods study by Pallin et al. from the United Kingdom surveyed 16 radiation therapists after completing an online nutrition course for living with and beyond cancer. It was found that radiation therapists self‐reported improvement in capability, opportunity and motivation to deliver advice on diet [40].

Additionally, incorporating lifestyle behaviour triage practices and support into undergraduate education could better prepare future radiation therapists for this aspect of patient care. Furthermore, radiation therapists need to have thorough knowledge of available referral resources and processes [36]. Combining enhanced education with improved referral links could reinforce radiation therapists' capacity to provide quality supportive care while maintaining an appropriate scope of practice.

### 4.4 Limitations

A limitation of this study is the recruitment of participants from only three States (ACT, NSW and QLD); however, this was a preliminary dissemination and it is intended that women with breast cancer will undertake the survey in the remaining States in the future. In addition, this survey design relied on self‐reporting, which could produce bias.

The current workforce shortage in RT may explain the limited response rate of this survey (*n* = 90) [41]. While it was carefully considered to have participants complete the survey on‐site to ensure suitable assistance from staff (before or after their treatment), it also added a layer of complexity to ensure staff could coordinate the time and facilities to do so. Lastly, the SDQs are a brief tool; despite being useful in describing a population's trends, they do not accurately represent individual data.

## 5 Conclusion

The findings from the study may be considered further evidence that women who are receiving RT for breast cancer could potentially improve their health outcomes with the implementation of dietary support. The study highlights the importance of identifying and addressing nutritional deficiencies in women with breast cancer, which can have a profound impact on their overall health and wellbeing. However, a revision of radiation therapists' education is required to effectively integrate dietary screening and support into breast cancer RT treatment regimens. By combining diet and nutrition as supportive care topics in the undergraduate curriculum and postgraduate learning opportunities (including understanding referral pathways) radiation therapists will be better positioned to meet the nutritional support needs identified in this research while maintaining an appropriate scope of practice.

## Conflicts of Interest

The authors declare no conflicts of interest.

## Supporting information


Appendix S1.

Appendix S2.

## Data Availability

Data available on request from the authors.

## References

[1] Australian Institute of Health and Welfare , “Diet,” 2023 Canberra [cited 2024 18th March], https://www.aihw.gov.au.

[2] Organization W H , Healthy Diet (World Health Organization, 2019).

[3] A. a. Alkerwi , “Diet Quality Concept,” Nutrition 30, no. 6 (2014): 613–618. 24800663 10.1016/j.nut.2013.10.001

[4] P. J. Skerrett and W. C. Willett , “Essentials of Healthy Eating: A Guide,” Journal of Midwifery & Women's Health 55, no. 6 (2010): 492–501. 10.1016/j.jmwh.2010.06.019 PMC3471136 20974411

[5] C. Katsura , I. Ogunmwonyi , H. K. Kankam , and S. Saha , “Breast Cancer: Presentation, Investigation and Management,” British Journal of Hospital Medicine 83, no. 2 (2022): 1–7. 10.12968/hmed.2021.0459 35243878

[6] B. Smolarz , A. Z. Nowak , and H. Romanowicz , “Breast Cancer—Epidemiology, Classification, Pathogenesis and Treatment (Review of Literature),” Cancers 14, no. 10 (2022): 2569. 35626173 10.3390/cancers14102569 PMC9139759

[7] H. S. Ng , D. Roder , B. Koczwara , and A. Vitry , “Comorbidity, Physical and Mental Health Among Cancer Patients and Survivors: An Australian Population‐Based Study,” Asia‐Pacific Journal of Clinical Oncology 14, no. 2 (2018): e181–e192. 28371441 10.1111/ajco.12677

[8] M. R. Fu , D. Axelrod , A. A. Guth , et al., “Comorbidities and Quality of Life Among Breast Cancer Survivors: A Prospective Study,” Journal of Personalized Medicine 5, no. 3 (2015): 229–242, 10.3390/jpm5030229. 26132751 PMC4600145

[9] K. P. Trayes and S. E. Cokenakes , “Breast Cancer Treatment,” American Family Physician 104, no. 2 (2021): 171–178. 34383430

[10] C. L. Shapiro and A. Recht , “Side Effects of Adjuvant Treatment of Breast Cancer,” New England Journal of Medicine 344, no. 26 (2001): 1997–2008. 11430330 10.1056/NEJM200106283442607

[11] M. Darooghegi Mofrad , F. Siassi , B. Guilani , N. Bellissimo , K. Suitor , and L. Azadbakht , “The Association of Food Quality Index With Mental Health in Women: A Cross‐Sectional Study,” BMC Research Notes 13, no. 1 (2020): 557. 33298144 10.1186/s13104-020-05401-x PMC7726882

[12] K. Davenport , J. E. Houston , and M. D. Griffiths , “Excessive Eating and Compulsive Buying Behaviours in Women: An Empirical Pilot Study Examining Reward Sensitivity, Anxiety, Impulsivity, Self‐Esteem and Social Desirability,” International Journal of Mental Health and Addiction 10, no. 4 (2012): 474–489.

[13] A. Hill , N. Kiss , B. Hodgson , T. C. Crowe , and A. D. Walsh , “Associations Between Nutritional Status, Weight Loss, Radiotherapy Treatment Toxicity and Treatment Outcomes in Gastrointestinal Cancer Patients,” Clinical Nutrition 30, no. 1 (2011): 92–98. 20719409 10.1016/j.clnu.2010.07.015

[14] F. Siddiqui and B. Movsas , eds., “Management of Radiation Toxicity in Head and Neck Cancers,” in Seminars in Radiation Oncology (Elsevier, 2017). 10.1016/j.semradonc.2017.04.008 28865517

[15] M. Taberna , F. Gil Moncayo , E. Jané‐Salas , et al., “The Multidisciplinary Team (MDT) Approach and Quality of Care,” Frontiers in Oncology 10 (2020): 85. 32266126 10.3389/fonc.2020.00085 PMC7100151

[16] A. S. J. R. Detsky , B. M , J. P. Baker , et al., “What is Subjective Global Assessment of Nutritional Status?,” Journal of Parenteral and Enteral Nutrition 11, no. 1 (1987): 8–13. 3820522 10.1177/014860718701100108

[17] E. Isenring , J. Hill , W. Davidson , et al., “Evidence Based Practice Guidelines for the Nutritional Management of Patients Receiving Radiation Therapy,” Nutrition and Dietetics 65, no. S1 (2008): 1–20.

[18] G. K. B. Halkett and L. J. Kristjanson , “Patients' Perspectives on the Role of Radiation Therapists,” Patient Education and Counseling 69, no. 1 (2007): 76–83. 17855042 10.1016/j.pec.2007.07.004

[19] B. L. Arnold , G. Halkett , H. Dhillon , and A. Girgis , “Do Radiation Therapists Feel Able to Routinely Screen for Symptoms and Distress in People With Cancer: Barriers Impacting Practice,” Journal of Medical Radiation Sciences 68, no. 2 (2021): 149–156. 33729701 10.1002/jmrs.465 PMC8168062

[20] K. Elsner , D. Naehrig , G. K. B. Halkett , and H. M. Dhillon , “Reduced Patient Anxiety as a Result of Radiation Therapist‐Led Psychosocial Support: A Systematic Review,” Journal of Medical Radiation Sciences 64, no. 3 (2017): 220–231. 28160448 10.1002/jmrs.208 PMC5587663

[21] Australian Bureau of Statistics , “National Nutrition Survey 1995, Australian Capitol Territory 2021.” [cited 2024 March 25th]. Available from: https://www.abs.gov.au/.

[22] G. C. Marks , K. Webb , I. H. Rutishauser , and M. Riley , “Monitoring Food Habits in the Australian Population Using Short Questions,” Canberra: Commonwealth of Australia 200 (2001): 1–112.

[23] E. L. James , F. Stacey , K. Chapman , et al., “Exercise and Nutrition Routine Improving Cancer Health (ENRICH): The Protocol for a Randomized Efficacy Trial of a Nutrition and Physical Activity Program for Adult Cancer Survivors and Carers,” BMC Public Health 11, no. 1 (2011): 1–9, 10.1186/1471-2458-11-236. 21496251 PMC3101179

[24] J. Bryant , B. Bonevski , C. L. Paul , and C. L. Lecathelinais , “A Cross‐Sectional Survey of Health Risk Behaviour Clusters Among a Sample of Socially Disadvantaged Australian Welfare Recipients,” Australian and New Zealand Journal of Public Health 37, no. 2 (2013): 118–123. 23551469 10.1111/1753-6405.12028

[25] K. Bush , D. R. Kivlahan , M. B. McDonell , S. D. Fihn , K. A. Bradley , and Project, f t A C Q I , “The AUDIT Alcohol Consumption Questions (AUDIT‐C): An Effective Brief Screening Test for Problem Drinking,” Archives of Internal Medicine 158, no. 16 (1998): 1789–1795. 9738608 10.1001/archinte.158.16.1789

[26] National Health and Medical Research Council , Australian Dietary Guidelines (National Health and Medical Research Council, 2013).

[27] Statistics, A B o , “Australian Health Survey: Nutrition First Results—Food and Nutrients Canberra,” 2011, https://www.abs.gov.au.

[28] P. G. Corr , W. Hudson , and N. Kalita , “Cancer Care and Nutrition Counseling: The Role of the Oncologist in Patient Learning and Behavior Change,” Global Advances in Integrative Medicine and Health 13 (2024): 27536130241285029. 39280090 10.1177/27536130241285029 PMC11402076

[29] Y.‐H. Chou , V. C.‐R. Hsieh , X. Chen , T.‐Y. Huang , and S.‐H. Shieh , “Unmet Supportive Care Needs of Survival Patients With Breast Cancer in Different Cancer Stages and Treatment Phases,” Taiwanese Journal of Obstetrics & Gynecology 59, no. 2 (2020): 231–236. 32127143 10.1016/j.tjog.2020.01.010

[30] J. D. Harrison , J. M. Young , M. A. Price , P. N. Butow , and M. J. Solomon , “What Are the Unmet Supportive Care Needs of People With Cancer? A Systematic Review,” Supportive Care in Cancer 17, no. 8 (2009): 1117–1128. 19319577 10.1007/s00520-009-0615-5

[31] C. Carter , J. E. Harnett , I. Krass , and I. C. Gelissen , “A Review of Primary Healthcare Practitioners' Views About Nutrition: Implications for Medical Education,” International Journal of Medical Education 13 (2022): 124–137. 35634903 10.5116/ijme.6271.3aa2 PMC9902177

[32] J. Crowley , L. Ball , and G. J. Hiddink , “Nutrition in Medical Education: A Systematic Review,” Lancet Planetary Health 3, no. 9 (2019): e379–e389. 31538623 10.1016/S2542-5196(19)30171-8

[33] M. Frenkel , K. J. Sapire , J. Lacey , C. Zollman , and V. S. Sierpina , “What Should I Eat?”—Addressing Questions and Challenges Related to Nutrition in the Integrative Oncology Setting,” Current Oncology Reports 24, no. 11 (2022): 1557–1567. 35788876 10.1007/s11912-022-01308-x

[34] E. A. Johnston , J. C. van der Pols , and S. Ekberg , “Needs, Preferences, and Experiences of Adult Cancer Survivors in Accessing Dietary Information Post‐Treatment: A Scoping Review,” European Journal of Cancer Care 30, no. 2 (2021): e13381. 33377564 10.1111/ecc.13381

[35] L. Feighan , L. MacDonald‐Wicks , R. Callister , and Y. Surjan , “Practitioner Perceptions on the Use of Exercise and Nutritional Interventions for Patients With Breast Cancer Receiving Radiation Therapy,” Journal of Medical Radiation Sciences 70, no. 4 (2023): 444–453. 37559550 10.1002/jmrs.713 PMC10715360

[36] A. Erlich , E. Posluns , E. Stokes , and L. Di Prospero , “Food for Thought: Are Radiation Therapists Able to Recognize Patients Who Would Benefit From Dietary Counseling?,” Journal of Medical Imaging and Radiation Sciences 46, no. 3 (2015): S13–S22. 31052102 10.1016/j.jmir.2015.04.017

[37] N. D. Pallin , R. J. Beeken , K. Pritchard‐Jones , L. Charlesworth , N. Woznitza , and A. Fisher , “Therapeutic Radiographers' Delivery of Health Behaviour Change Advice to Those Living With and Beyond Cancer: A Qualitative Study,” BMJ Open 10, no. 8 (2020): e039909. 10.1136/bmjopen-2020-039909 PMC7422652 32788193

[38] N. D. Pallin , R. J. Beeken , K. P. Jones , N. Woznitza , and A. Fisher , “A Survey of Therapeutic Radiographers' Knowledge, Practices, and Barriers in Delivering Health Behaviour Advice to Cancer Patients,” Journal of Cancer Education 37, no. 4 (2022): 890–897. 33063254 10.1007/s13187-020-01896-x PMC9399055

[39] K. Konstantinidis , I. Apostolakis , and P. Karaiskos , “A Narrative Review of e‐Learning in Professional Education of Healthcare Professionals in Medical Imaging and Radiation Therapy,” Radiography 28, no. 2 (2022): 565–570. 34937680 10.1016/j.radi.2021.12.002

[40] N. Pallin , J. Webb , L. Brown , et al., “Online Training Resources to Aid Therapeutic Radiographers in Engaging in Conversations About Physical Activity and Diet: A Mixed Methods Study,” Radiography 28, no. 1 (2022): 124–132, 10.1016/j.radi.2021.09.004. 34583887

[41] Australian Government , “Jobs and Skills Australia Canberra,” 2024, https://www.jobsandskills.gov.au/.

