# Supplementary material for: Fuelling Recovery: Is There a Role for Radiation Therapists in Optimising Nutrition for Women With Breast Cancer?
Source: J Med Radiat Sci. 2025 Mar 27;72(3):350–60. doi: 10.1002/jmrs.874 (PMC12420669; doi:10.1002/jmrs.874)
Supplement: Supplementary file 1 — Appendix S1. [file JMRS-72-350-s002.pdf]

The following questions are about your eating habits on a typical day or in typical week.
(Circle one number only for each question)

16. Including snacks, how many times do you usually have something to eat in a day, including evenings?

Once	1
2 to 4 times	2
5 to 6 times	3
7 or more times	4
Don't know/varies	5

17. How many days per week do you usually have something to eat for breakfast?

Rarely or never	1
1 to 2 days	2
3 to 4 days	3
5 or more days	4
Don't know/varies	5

22. A serving of vegetables is 75 grams (e.g. 1/2 cup of cooked vegetables, 1 cup of salad, or one medium sized potato). How many serves of vegetables do you usually eat each day?

1 serve	1
2 serves	2
3 serves	3
4 serves	4
5 serves	5
6 serves or more	6
Less than one serve	7
Don't eat vegetables	8

23. How often do you eat salad? (Salad includes mixed green salad and other mixtures of raw vegetables)

Less than once per week	1
1-2 times per week	2
3-7 times per week	3
7 or more times per week	4

24. Not counting potatoes and salad, how often do you eat cooked vegetables?

Less than once per week	1
1-2 times per week	2
3-7 times per week	3
7 or more times per week	4

25. How often do you eat potatoes? (<u>not</u> including chips, French fries, wedges, fried potatoes, or crisps)

Less than once per week	1
1-2 times per week	2
3-7 times per week	3
7 or more times per week	4

26. How often do you eat chips, French fries, wedges, fried potatoes, or crisps?

Rarely or never	0
Less than once per week	1
1-2 times per week	2
3-7 times per week	3
7 or more times per week	4

27. A serving of fruit is 125 grams (e.g. one medium piece of fruit, two small pieces of fruit, one cup of chopped, frozen or canned fruit, <u>or</u> 2 tablespoons of dried fruit). How many serves of fruit do you usually eat each day?

1 serve	1
2 serves	2
3 serves	3
4 serves	4
5 serves	5
6 serves	6
Less than one serve	7
Don't eat fruit	8

28. How often do you drink fruit juices such as orange, grapefruit, or tomato?

Less than once per week	1
1-2 times per week	2
3-7 times per week	3
7 or more times per week	4

29. Not counting juice, how often do you eat fruit? (Includes fresh, canned, frozen, and dried)

Less than once per week	1
1-2 times per week	2
3-7 times per week	3
7 or more times per week	4

30. How often do you eat bread? (includes bread, rolls, flat breads, crumpets, bagels, English or bread type muffins)

Less than once per day	1
1-2 times per day	2
2-4 times per day	3
4 or more times per day	4

31. How often do you eat breakfast cereal? (ready-made, home-made, or cooked)

Rarely or never	0
1-2 times per week	1
3 -7 times per week	2
7 or more times per week	3

32. How often do you eat pasta, rice noodles, or other cooked cereals? (not including cooked breakfast cereal)

Rarely or never	0
1-2 times per week	1
3-7 times per week	2
7 or more times per week	3

33. How often do you eat meat products such as sausages, frankfurters, devon, salami, meat pies, bacon, or ham?

Rarely or never	0
1-2 times per week	1
3-7 times per week	2
7 or more times per week	3

34. How often do you eat red meat? (beef, lamb, liver and kidney but <u>not</u> pork or ham)

Rarely or never	0
1-2 times per week	1
3-7 times per week	2
7 or more times per week	3

35. What type of milk do you usually consume?

Whole milk	1
Low/reduced fat	2
Skim milk	3
Evaporated or sweetened milk	4
None of the above	5
Both low fat and skim milk	6
Both whole and low fat milk	7
Both whole and skim	8
Non-dairy milk	9

36. How much milk (in total) do you usually consume in a day?

Less than 150mL	1
150-300mL	2
301-600mL	3
More than 600mL	4

37. How often do you eat biscuits, cakes, pastries, confectionary, and sugar-sweetened soft drinks or cordials?

Rarely or never	0
1-2 times per week	1
3-7 times per week	2
7 or more times per week	3
