# Supplementary material for: Fuelling Recovery: Is There a Role for Radiation Therapists in Optimising Nutrition for Women With Breast Cancer?
Source: J Med Radiat Sci. 2025 Mar 27;72(3):350–60. doi: 10.1002/jmrs.874 (PMC12420669; doi:10.1002/jmrs.874)
Supplement: Supplementary file 2 — Appendix S2. [file JMRS-72-350-s001.pdf]

THE UNIVERSITY OF NEWCASTLE AUSTRALIA

GLOBAL CENTRE FOR RESEARCH AND TRAINING IN RADIATION ONCOLOGY

Survey for Patients with Breast Cancer receiving Radiation Therapy

Thank you for your participation in this survey. The survey will take approx. 20 minutes to complete. You will need to answer all questions and your answers will be saved automatically. Thank you!

Section A: Demographics

* 1. What is your age in years?

- ◯ 18 - 24
- ◯ 25 - 34
- ◯ 35 - 44
- ◯ 45 - 54
- ◯ 55 - 64
- ◯ 65 - 74
- ◯ 75 - 84
- ◯ 85 or older

* 2. What is your postcode?

* 3. What is your marital status?

- Married
- Widowed
- Divorced/Separated
- De Facto
- Never married

* 4. Do you have carer responsibilities?
(A carer is anyone who cares, unpaid, for a friend or family member who due to age, illness, disability, a mental health problem, or an addiction, cannot cope without their support)

- Yes
- No

* 5. Who do you care for?

- Children (own or fostered)
- Spouse/partner
- Disabled family member/s
- Sibling/s
- Parent/s
- Friend/s

QuestionPro

6. Please select from the following options for which best describes your treatment for breast cancer (you may choose more than one).

	Please select
I have had a lumpectomy (only a portion of the breast was removed)	☐
I have had a mastectomy (the whole breast was removed)	☐
I have had a breast reconstruction after a mastectomy	☐
I will have a breast reconstruction at a later date	☐
I received chemotherapy before radiation therapy	☐
I am receiving Herceptin (targeted therapy) alongside radiation therapy	☐
I have received a sentinal node biopsy	☐
I have a received an axillary dissection	☐

* 7. Do you currently have lymphoedema (swelling of the hand or arm after breast cancer treatment)?

○ Yes

○ No

* 8. Please select which best describes your lymphoedema

○ No problem: no noticeable swelling

○ Mild lymphoedema: soft swelling that is not obvious to others, which comes and goes

○ Moderate lymphoedema: swelling with occasional hardness in some areas that is obvious to others and is always present

○ Severe lymphoedema: profuse swelling with thickened skin, constant hardness and a very large, heavy arm that is extremely obvious to others and is always present

* 9. Do you wear a compression garment for lymphoedema?

- ◯ No
- ◯ Yes, a sleeve
- ◯ Yes, a sleeve and glove

* 10. Has any professional given you information about lymphoedema?

- ◯ Yes
- ◯ No

* 11. Who gave you information about lymphoedema?

- ☐ Surgeon
- ☐ Surgical nurse
- ☐ Radiation oncologist
- ☐ Radiation nurse
- ☐ Radiation therapist
- ☐ Medical oncologist
- ☐ Medical oncology nurse
- ☐ My own reading/investigation
- ☐ My general practitioner
- ☐ Other

If selected 'other' please specify:

* 12. Have you had a baseline lymphoedema check?

- Yes, using L-Dex (bioimpedence with U400 or SOZO)
- Yes, using tape measure
- Yes, other
- No

If selected 'other' please specify:

* 13. How many days in total of Radiation Therapy are you to receive? (Please ask staff if unsure)

* 14. How many days of Radiation Therapy have you received to date? (Please ask staff if unsure)

15. Have you ever had, or do you have any of the following? (Please select multiple answers if required)

- High blood pressure (hypertension)
- High cholesterol/triglycerides (lipid problems)
- Liver/kidney condition
- Diabetes (Type 1)
- Diabetes (Type 2)

- [ ] Heart condition (heart attack, coronary myocardial infarction, angina pectoris)
- [ ] Stroke
- [ ] Lung conditions (asthma, chronic bronchitis, emphysema of the lungs)
- [ ] Musculoskeletal disorders (osteoporosis, back problems)
- [ ] Arthritis (osteoarthritis/rheumatoid arthritis)
- [ ] Stomach or duodenal ulcer
- [ ] Chronic headaches/migraine
- [ ] Anxiety, depression, post traumatic stress disorder
- [ ] None of the above
- [ ] Other

If selected 'other' please specify:

Please indicate any medications/treatments you are receiving for the preceding condition/s

* 16. What is your current employment status?

- ( ) Employed full time
- ( ) Employed part time
- ( ) Casual
- ( ) Self-employed
- ( ) On maternity leave
- ( ) Full time parent/carer

- Unemployed - looking for work
- Retired
- Volunteer

* 17. Has the number of hours you work changed since commencing cancer treatment?

- Yes
- No

* 18. Which describes your changes in work hours? Please select from the drop down menu.

- I work more hours since commencing cancer treatment
- I work less hours since commencing cancer treatment
- Other

If selected 'other' please specify:

19. Please describe the reason for your change in work hours:

Section B: General Health - Before Cancer Diagnosis

For each of the following questions, please select the answer that best describes your general health **BEFORE YOUR CANCER DIAGNOSIS.**

* 1. **Before your cancer diagnosis,** in general, would you say your health was

- Excellent
- Very good
- Good
- Fair
- Poor

* 2. The following questions are about activities you might have done during a typical day.
Before your cancer diagnosis, did your health limit you in these activities. If so, how much?

	Yes, limited a lot	Yes, limited a little	No, not limited at all
a. **Moderate activities**, such as move a table, pushing a vacuum cleaner, bowling or playing golf	○	○	○
Climbing **several** flights of stairs	○	○	○

* 3. **Before your cancer diagnosis,** how often did you have any of the following problems with your work or other regular daily activities **as a result of your physical health?**

	All of the time	Most of the time	Some of the time	A little of the time	None of the time
a. **Accomplished less** than you would like	○	○	○	○	○
b. Were limited in the **kind** of work or other activities	○	○	○	○	○

* 4. **Before your cancer diagnosis,** how often did you have any of the following problems with your work or other regular daily activities as a result of any emotional problems (such as feeling depressed or anxious)?

	All of the time	Most of the time	Some of the time	A little of the time	None of the time

a. **Accomplished less** than you would like	○	○	○	○	○
b. Did work or other activities **less carefully than usual**	○	○	○	○	○

* 5. **Before your cancer diagnosis,** how often did pain interfere with your normal work (including both work outside the home and housework)?

- ○ Not at all
- ○ A little bit
- ○ Moderately
- ○ Quite a bit
- ○ Extremely

* 6. For each question, please give the one answer that comes closest to the way you felt.
Before your cancer diagnosis, how often...

	All of the time	Most of the time	Some of the time	A little of the time	None of the time
a. Did you feel calm and peaceful?	○	○	○	○	○
b. Did you have a lot of energy?	○	○	○	○	○
c. Did you feel downhearted and depressed?	○	○	○	○	○

* 7. **Before your cancer diagnosis,** how often did your physical health or emotional problems interfere with your social activities (like visiting with friends, relatives, etc.)?

- ○ All of the time
- ○ Most of the time
- ○ Some of the time
- ○ A little of the time

QuestionPro

◯ None of the time

Section C: General Health - Currently

For each of the following questions, please select the answers that best describe your general health **CURRENTLY.**

* 1. **Currently,** in general, would you say your health is:

◯ Excellent

◯ Very good

◯ Good

◯ Fair

◯ Poor

* 2. The following questions are about activities you might do during a typical day. **Currently**, does **your health limit you** in these activities? If so, how much?

	Yes, limited a lot	Yes, limited a little (2)	No, not limited at all
a. **Moderate activities**, such as moving a table, pushing a vacuum cleaner, bowling, or playing golf	◯	◯	◯
b. Climbing **several** flights of stairs	◯	◯	◯

* 3. **Currently,** how much time do you have with any of the following problems with your work or other regular daily activities **as a result of your physical health?**

	All of the time	Most of the time	Some of the time	A little of the time	None of the time
a. **Accomplished less** than you would like	◯	◯	◯	◯	◯

b. Were limited in the **kind** of work or other activities	◯	◯	◯	◯	◯

* 4. **Currently,** how much time do you have with any of the following problems with your work or other regular daily activities **as a result of any emotional problems** (such as feeling depressed or anxious)?

	All of the time	Most of the time	Some of the time	A little of the time	None of the time
a. **Accomplished less** than you would like	◯	◯	◯	◯	◯
Did work or other activities less carefully than usual	◯	◯	◯	◯	◯

* 5. **Currently,** how much does **pain** interfere with your normal work (including both work outside the home and housework)

- ◯ Not at all
- ◯ A little bit
- ◯ Moderately
- ◯ Quite a bit
- ◯ Extremely

* 6. For each question, please give the one answer that comes closest to the way you feel.
Currently, how much of the time...

	All of the time	Most of the time	Some of the time	A little of the time	None of the time
a. Do you feel calm and peaceful?	◯	◯	◯	◯	◯
b. Do you have a lot of energy?	◯	◯	◯	◯	◯
Do you feel downhearted and depressed?	◯	◯	◯	◯	◯

* 7. **Currently,** how much of the time does your physical health or emotional problems interfere with your social activities (like visiting with friends, relatives, etc.)?

- All of the time
- Most of the time
- Some of the time
- A little of the time
- None of the time

* 8. Do you currently smoke any tobacco products?

- Daily
- At least once a week
- Less often than once a week
- Not at all

* 9. Have you smoked at least 100 cigarettes or the equivalent amount of tobacco in your life?

- Yes
- No
- Don't know

* 10. How often do you have a drink containing alcohol?

- Never
- Monthly or less
- 2-4 times a month

- ◯ 2-3 times a week
- ◯ 4 or more times a week

* 11. How many standard drinks containing alcohol do you have on a typical day?

- ◯ 1 or 2
- ◯ 3 or 4
- ◯ 5 or 6
- ◯ 7 to 9
- ◯ 10 or more

* 12. How often do you have six or more drinks on one occasion?

- ◯ Never
- ◯ Less than monthly
- ◯ Monthly
- ◯ Weekly
- ◯ Daily or almost daily

Section D: Physical Activity - Before Cancer Diagnosis

For each of the following questions, please select the answers with regard to **BEFORE YOUR CANCER DIAGNOSIS.**

The next two questions are about the amount of physical activity you did **BEFORE YOUR CANCER DIAGNOSIS**

1. How many times did you do each type of activity **PER WEEK?** Only count the number of times when the activity lasted for 10 minutes or more.
(If you did not do an activity, please write "0").

* Walk briskly (for recreational or exercise, or to get from place to place)

times per week

* Moderate leisure activity (like social tennis, moderate exercise classes, recreational swimming, dancing)

times per week

* Vigorous leisure activity (that makes you breathe harder or puff and pant like aerobics, competitive sport, vigorous cycling, running, swimming)

timers per week

* Vigorous household or garden chores (that make you breathe harder or puff and pant)

times per week

* 2. If you **add up** all the times you spent doing each activity **PER WEEK**, how much time did you spend **ALTOGETHER** doing each type of activity? (If you did not do an activity, please write "0").

	Hours	Minutes
Walking briskly (for recreation or exercise, or to get from place to place)		

QuestionPro

Moderate leisure activities (like social tennis, moderate exercise classes, recreational swimming, dancing)		
Vigorous leisure activity (that makes you breathe harder or puff and pant like aerobics, competitive sport, vigorous cycling, running, swimming)		
Vigorous household or garden chores (that make you breathe harder or puff and pant)		

The next two questions are about the amount of resistance training you did **BEFORE YOUR CANCER DIAGNOSIS**.

Examples of resistance training may include: using a Gymstick, lifting or pushing dumbbells and/or barbells, using elastic exercise bands or TheraBand's, using your own weight as resistance (e.g. pushups, squats, lunges, leg lifts, stomach crunches), weight machines.

* 3. How many times **PER WEEK** did you do any resistance training? Only count the number of times when the activity lasted for 10 minutes or more.
(If you did not do any, please write "0")

times per week

* 4. If you **add up** all the times you spent doing resistance training **PER WEEK**, how much time did you spend **ALTOGETHER** doing resistance training? (If you did not do any, please write "0").

	Hours	Minutes
Resistance training		

Section D: Physical Activity - Currently

For each of the following questions, please take note of the **timeframe** - e.g. 'IN THE LAST WEEK' or 'IN THE NEXT 8 WEEKS'.

The next two questions are about the amount of physical activity you did **IN THE LAST WEEK**

1. How many times did you do each type of activity **IN THE LAST WEEK?** Only count the number of times when the activity lasted for 10 minutes or more.

(If you did not do an activity, please write "0").

* Walk briskly (for recreational or exercise, or to get from place to place)

times in the last week

* Moderate leisure activity (like social tennis, moderate exercise classes, recreational swimming, dancing)

times in the last week

* Vigorous leisure activity (that makes you breathe harder or puff and pant like aerobics, competitive sport, vigorous cycling, running, swimming)

times in the last week

* Vigorous household or garden chores (that make you breathe harder or puff and pant)

times in the last week

* The next few questions are about regular physical activity. Regular physical activity is defined as achieving at least 30 minutes of moderate or vigorous-intensity activity on most, preferably all, days of the week.

2. How likely is it that you will do regular physical activity **IN THE NEXT 8 WEEKS?**

- ◯ 10%
- ◯ 20%
- ◯ 30%
- ◯ 40%
- ◯ 50%
- ◯ 60%
- ◯ 70%
- ◯ 80%
- ◯ 90%
- ◯ 100%

The next questions as you to rate how confident you are that you could participant in regular physical activity over the **OVER THE NEXT 8 WEEKS**. Please rate how much the following circumstances affect your confidence to participate in regular physical activity over the next 8 weeks.
If you do not plan on doing regular physical activity select 'not confident at all'.

* 3. How confident are you that you could participate in regular physical activity **OVER THE NEXT 8 WEEKS?**

	Not confident at all	Not very confident	Moderately confident	Very confident	Extremely confident

When I am a little tired:	○	○	○	○	○
When I am in a bad mood or feeling depressed:	○	○	○	○	○
When I have to do it by myself:	○	○	○	○	○
When it becomes boring:	○	○	○	○	○
When I can't notice any improvements in my fitness:	○	○	○	○	○
When I have many other demands on my time:	○	○	○	○	○
When I feel a little stiff and sore:	○	○	○	○	○
When the weather is bad:	○	○	○	○	○
When I have to get up early, even on weekends:	○	○	○	○	○

* 4. To what extent do you agree or disagree that participating in regular physical activity **OVER THE NEXT 8 WEEKS** would do for you:

Help me reduce tension or manage stress:

I would feel more confident about my health by doing regular physical activity:

I would sleep better:

Regular physical activity would take too much of my time:

QuestionPro

I would have less time for my family and friends if I participated in regular physical activity:

I'd be too tired to do regular physical activity because of my other daily responsibilities:

Regular physical activity would help me have a more positive outlook:

Regular physical activity would help me control my weight:

I'd worry about looking awkward if others say me doing regular physical activity:

Participating in regular physical activity would cost too much money:

* 5. **OVER THE NEXT 8 WEEKS**, how much are people in your social network likely to:

QuestionPro

Help me to participate in regular physical activity:

Provide me with support I need in order to do regular physical activity:

* 6. How much do you weigh (without shoes)?

kilograms

* 7. How tall are you (without shoes)?

centimetres

Section E: Diet

The following questions are regarding your diet.

* 1. Including snacks, how many times do you usually have something to eat in a day? (including evenings)

.

BEFORE your cancer diagnosis

-- Select --

CURRENTLY

-- Select --

* 2. How many days per week do you usually have something to eat for breakfast?

.

BEFORE your cancer diagnosis
-- Select --

CURRENTLY
-- Select --

* 3. A serving of vegetables is 75 grams (e.g. half a cup of cooked vegetables, 1 cup of salad, one medium sized potato).How many serves of vegetables do you usually eat each day?

.

BEFORE your cancer diagnosis
-- Select --

CURRENTLY
-- Select --

* 4. How often do you eat salad? (Salad includes mixed green salad and other mixtures of raw vegetables)

.

BEFORE your cancer diagnosis
-- Select --

CURRENTLY
-- Select --

* 5. Not counting potatoes and salad, how often do you eat cooked vegetables?

.

BEFORE your cancer diagnosis
-- Select --

CURRENTLY
-- Select --

* 6. How often do you eat potatoes? (NOT including chips, french fries, wedges, fried potato or crisps)

.

BEFORE your diagnosis
-- Select --

CURRENTLY
-- Select --

* 7. How often do you eat chips, french fries, wedges, fried potato or crisps

.

BEFORE your cancer diagnosis
-- Select --

CURRENTLY
-- Select --

* 8. A serving of fruit is 125 grams (e.g. one medium piece of fruit, two small pieces of fruit, one cup of chopped, frozen or canned fruit, or 2 tablespoons of dried fruit).

.

BEFORE your cancer diagnosis
-- Select --

CURRENTLY
-- Select --

* 9. How often do you drink fruit juices such as orange, grapefruit, or tomato?

.

BEFORE your cancer diagnosis
-- Select --

CURRENTLY
-- Select --

* 10. Not counting juice, how often do you eat fruit? (includes fresh, canned, frozen and dried)

.

BEFORE your cancer diagnosis

-- Select --

CURRENTLY

-- Select --

* 11. How often do you eat bread? (includes bread, rolls, flat breads, crumpets, bagels, English or bread type muffins)

.

BEFORE your cancer diagnosis

-- Select --

CURRENTLY

-- Select --

* 12. What type of bread do you usually eat?

.

BEFORE your cancer diagnosis

-- Select --

CURRENTLY

-- Select --

* 13. How often do you eat breakfast cereal? (ready-made, home made or cooked)

.

BEFORE your cancer diagnosis

-- Select --

CURRENTLY

-- Select --

* 14. How often do you eat pasta, rice, noodles or other cooked cereals? (not including cooked breakfast cereal)

.

BEFORE your cancer diagnosis
-- Select --

CURRENTLY
-- Select --

* 15. How often do you eat meat products such as sausages, frankfurters, devon, salami, meat pies, bacon or ham?

.

BEFORE your cancer diagnosis
-- Select --

CURRENTLY
-- Select --

* 16. How often do you eat red meat? (Beef, lamb and pork)

.

BEFORE your cancer diagnosis
-- Select --

CURRENTLY
-- Select --

* 17. What type of milk do you usually consume? You may choose more than one answer.

.

BEFORE your cancer diagnosis
-- Select --

CURRENTLY
-- Select --

* 18. How often do you eat biscuits, cakes, pastries, confectionery and sugar sweetened soft drinks or cordials?

.

QuestionPro

BEFORE your diagnosis

-- Select --

CURRENTLY

-- Select --

Section F: Exercise & Nutrition Program

This research seeks to investigate the role of exercise and nutrition during radiation therapy treatment for breast cancer in view of its demonstrated benefits in promoting and supporting health in other diseases. There is substantial evidence surrounding the benefit of exercise and nutrition on general health-related quality of life, however there is scope for investigation in the specific area of exercise and nutrition directly affecting the health-related quality of life of breast cancer patients undergoing radiation therapy treatment. Based on research conducted in other health areas, it might be assumed that exercise and nutrition would significantly benefit the health-related quality of life of patients with breast cancer undergoing radiation therapy treatment, if tailored to suit their specific needs.

The following questions are regarding your thoughts and feelings towards a tailored exercise and nutrition program if it were offered to you during your radiation therapy treatment, to better understand the feasibility of implementing such a program for future patients.

* 1. Please select an answer that best describes your feelings toward the following statements:

	Strongly agree	Agree	Somewhat agree	Neither agree or disagree	Somewhat disagree	Disagree	Strongly disagree
a. I am knowledgeable in the area of exercise and physical activity	○	○	○	○	○	○	○
b. I am knowledgeable in the area of nutrition and diet	○	○	○	○	○	○	○
c. During my radiation therapy treatment I have received sufficient information on exercise and physical activity as an intervention	○	○	○	○	○	○	○
d. During my radiation treatment I have received sufficient information on nutrition and diet as an intervention	○	○	○	○	○	○	○

* 2. How confident are you in undertaking your own exercise regimen during your radiation therapy treatment?

	Very confident	Somewhat confident	Confident	Slightly confident	Not confident
.	◯	◯	◯	◯	◯

* 3. How confident are you in maintaining a healthy diet during your radiation therapy treatment?

	Very confident	Somewhat confident	Confident	Slightly confident	Not confident
.	◯	◯	◯	◯	◯

* 4. Would you undertake a tailored exercise and nutrition program in your current position, if it were offered?

◯ Yes

◯ No

If No, please state why:

5. If your answer is Yes - what is your reasoning to undertake a tailored exercise and nutrition program? You may choose more than one answer.

☐ I need assistance to exercise and eat healthily

☐ I enjoy exercising

☐ I think it would help me through my radiation treatment

☐ I have been encouraged by staff to exercise and eat healthily

☐ Other - please specify:

Other - please specify:_comment

* 6. If you were to undertake a tailored exercise and nutrition program, how would you prefer it to be executed?

- [ ] Face-to-face consultation with a professional
- [ ] On the internet or via an 'app'
- [ ] Through video conference consultation with a professional
- [ ] In a group based program
- [ ] In a solo based program
- [ ] Other - please specify:

Other - please specify:_comment

* 7. How many hours per week would you commit to engaging in a tailored exercise and nutrition program?

- ○ Less than 2
- ○ 3-4
- ○ 5-6
- ○ More than 6
- ○ As many as are advised
